# Draft Genome Sequence of *Aeromonas veronii* Hm21, a Symbiotic Isolate from the Medicinal Leech Digestive Tract

**DOI:** 10.1128/genomeA.00800-13

**Published:** 2013-10-03

**Authors:** Lindsey Bomar, W. Zac Stephens, Michael C. Nelson, Katrina Velle, Karen Guillemin, Joerg Graf

**Affiliations:** Department of Molecular and Cell Biology, University of Connecticut, Storrs, Connecticut, USAa; Institute of Molecular Biology, University of Oregon, Eugene, Oregon, USAb

## Abstract

*Aeromonas veronii* strain Hm21 was isolated from the digestive tract of the medicinal leech *Hirudo verbana* and has been used to identify genes that are important for host colonization. This species is also a symbiont in the gut of zebrafish and is a pathogen of mammals and fish. We present here a 4.68-Mbp draft genome sequence for Hm21.

## GENOME ANNOUNCEMENT

*Aeromonas veronii* exhibits a dual lifestyle as a digestive tract symbiont of the medicinal leech *Hirudo verbana* (1, 2) and the zebrafish *Danio rerio* (3, 4) and also as a pathogen of fish (5) and people (6). *A. veronii* is virulent in mouse, fish, and waxworm septicemia models (7–9). The leech isolate, *A. veronii* Hm21, has also been used to identify genes that are important for the colonization of the leech digestive tract (10–12), which has revealed new information about host-microbe interactions (2, 13, 14). We present a draft genome of *A. veronii* Hm21.

Multiple platforms were used for sequencing the genome of Hm21: Sanger sequencing (6,570 reads), 454 pyrosequencing (71,004 reads), and Illumina GAII (29,913,618 reads), and these were assembled using the CLC Genomics Workbench (Aahrus, Denmark), yielding 75 contigs with a length of >2 kb. Multiple rRNA operons containing variants of the 16S and 23S rRNA genes and the presence of multiple transposable elements complicated the assembly (15, 16). The assembly was improved by Illumina sequencing of Nextera libraries from Hm21R (1) and fosmids from the Hm21 genome. Evidence from the Mauve alignments and breseq analysis were used to correct errors and bridge contigs (17). These improvements reduced the number of contigs to 50 (>2 kb) and increased the N_50_ value to 179,631 bp. The updated contigs totaled 4,684,957 bp at >200× coverage. RAST (18, 19) annotated 4,245 open reading frames and 96 RNAs. The G+C content is 58.7%.

The genome of Hm21 encodes several potential colonization and virulence factors. Most prominent are the presence of two type III secretion system (T3SSs), one of which, T3SS-1, is required for the colonization of the leech gut and for virulence in a mouse model (8). The recently sequenced *A. veronii* strain B565 does not possess a T3SS and was isolated from the sediments of a fish pond (20). Hm21 also possesses a type VI secretion system that has been shown in *Aeromonas hydrophila* to be important in virulence (21).

The genome sequence also sheds light on the microorganism’s metabolic potential. In addition to the central carbohydrate metabolism pathways, the genome of Hm21 encodes alternative nutrient utilization mechanisms. The genome encodes a sialic acid utilization pathway that is absent in the sequenced B565 strain; genes for an arginine deiminase pathway, several ABC-type transporters, and phosphotransferase system components are also present, suggesting that Hm21 can utilize amino acids and carbohydrates.

In contrast to *A. hydrophila* (15), *A. veronii* contains several mobile genetic elements, a genomic architecture that is more similar to *Aeromonas salmonicida* (22) and *Aeromonas caviae* (23). The genome contains at least two prophage loci and several insertion sequences (IS*630*, IS*5*, and ISDuV). The genome of *A. veronii* Hm21 revealed shared genes with other sequenced aeromonads and unique genes that may allow this strain to proliferate in a digestive tract and, under certain circumstances, to potentiate disease.

### Nucleotide sequence accession numbers.

This whole-genome shotgun project has been deposited at DDBJ/EMBL/GenBank under the accession no. ATFB00000000. The version described in this paper is version ATFB01000000.
